# Microwave Radiation and the Brain: Mechanisms, Current Status, and Future Prospects

**DOI:** 10.3390/ijms23169288

**Published:** 2022-08-18

**Authors:** Sohail Mumtaz, Juie Nahushkumar Rana, Eun Ha Choi, Ihn Han

**Affiliations:** 1 Department of Electrical and Biological Physics, Kwangwoon University, Seoul 01897, Korea; 2 Plasma Bioscience Research Center (PBRC), Applied Plasma Medicine Cencer, Kwangwoon University, Seoul 01897, Korea; 3 Department of Plasma Bio-Display, Kwangwoon University, Seoul 01897, Korea

**Keywords:** microwaves biological effects, cellular effects, radiations and brain, nonthermal plasma, flavonoids, Alzheimer’s disease, tissue damage, human health

## Abstract

Modern humanity wades daily through various radiations, resulting in frequent exposure and causing potentially important biological effects. Among them, the brain is the organ most sensitive to electromagnetic radiation (EMR) exposure. Despite numerous correlated studies, critical unknowns surround the different parameters used, including operational frequency, power density (i.e., energy dose), and irradiation time that could permit reproducibility and comparability between analyses. Furthermore, the interactions of EMR with biological systems and its precise mechanisms remain poorly characterized. In this review, recent approaches examining the effects of microwave radiations on the brain, specifically learning and memory capabilities, as well as the mechanisms of brain dysfunction with exposure as reported in the literature, are analyzed and interpreted to provide prospective views for future research directed at this important and novel medical technology for developing preventive and therapeutic strategies on brain degeneration caused by microwave radiation. Additionally, the interactions of microwaves with biological systems and possible mechanisms are presented in this review. Treatment with natural products and safe techniques to reduce harm to organs have become essential components of daily life, and some promising techniques to treat cancers and their radioprotective effects are summarized as well. This review can serve as a platform for researchers to understand the mechanism and interactions of microwave radiation with biological systems, the present scenario, and prospects for future studies on the effect of microwaves on the brain.

## 1. Introduction

Microwaves are recognized as nonionizing radiation, with a broad frequency spectrum ranging from 300 MHz to 300 GHz. Specifically, the bands from 300 MHz to 3 GHz are UHF (ultra-high-frequency), 3–30 GHz are SHF (super-high-frequency), and those from 30 to 300 GHz are EHF (extremely high-frequency). Additionally, microwaves that exceed a peak power of 100 MW with an operation frequency lying between 1 to 300 GHz are generally considered high-power microwaves (HPMs). HPMs are commonly employed in modern technologies, and have been proven to be indispensable in our lives, with uses in the commercial, military, and medical fields [1,2,3,4]. Pulse microwave sources and non-pulse microwave sources are the two types of HPM sources available. A microwave source with a rising edge of sub-nanoseconds or picoseconds is referred to as a pulsed microwave source. A primary drive (explosive or pulse generation system), a pulse compression system, a microwave generation source, and an antenna are all included. Through low-speed storage and fast release of energy, pulse sources typically transform energy into short-pulse EMR. Various HPM sources exist to meet the demand for HPM in applications, with the majority of them undergoing research to increase conversion efficiency. The generation of HPMs also represents an active area of research [5,6,7,8,9,10,11,12,13,14,15]. HPM has arisen as a new technology that provides a wide range of original applications while also providing innovative methods and changes to existing technologies from previous decades. HPMs can be used for a variety of future applications and to improve the performance of existing technologies in our daily lives. Further, progress in radio-frequency microwaves, among others, has provided novel diagnostic and therapeutic methods; whereas microwaves between 400 kHz to 10 GHz are presently being studied for their therapeutic purposes in the medical field [16,17,18], and have been explored for diagnostic applications, such as early-stage cancer and tumor detection, organ imaging, etc. [18,19,20,21,22,23,24,25,26,27].

Radio waves are electromagnetic waves that range from 3 kHz to 300 GHz in frequency. To observe astronomical objects, radio waves are commonly employed as envelope signals on radio communication and wavelength channels. Microwaves are a form of short-frequency radio waves. They can be categorized as a radio wave subclass. Microwaves have a frequency range of 300 MHz to 300 GHz. Microwaves are commonly employed in microwave ovens because resonance frequency of water molecules is in the microwave range. Radars, astronomy, navigation, and spectroscopy all employ microwaves. Accordingly, the development of advanced electronics and novel microwave-based systems has made microwaves an important part of our daily lives. More specifically, the increasing number of radio-wave-based applications has led to the investigation of their biological effects. In the following review, the possible mechanisms and interactions of radiations with biological systems are synthesized.

### 1.1. Interactions of Biological Systems with Electromagnetic Radiation (EMR)

**Figure 1:**
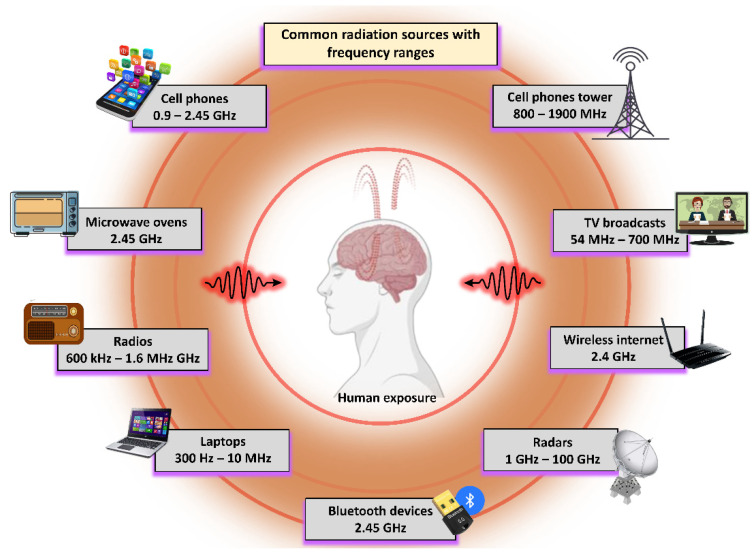
Depiction of daily commercial and household radiation sources with corresponding operational frequency ranges.

**Figure 2:**
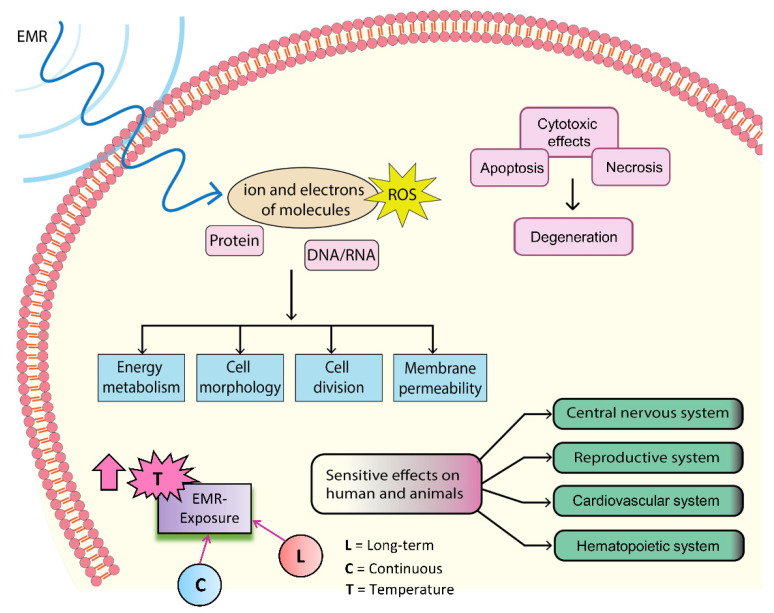
A generalized representation of the interactions between EMR and biological systems, with common effects. The EMR has an impact on molecular ions and electrons, as well as ROS, protein, and DNA/RNA levels. Furthermore, the EMR has cytotoxic effects on cells by causing degeneration, apoptosis, and necrosis. EMR has a strong impact on the central nervous system, reproductive system, cardiovascular system, and hematological system. Furthermore, the constant and long-term exposure of EMR to a biological system raises tissue temperature, which is a frequent effect of different stimuli.

Owing to the existence of various types of radiation in our daily living environments, the effects of biological system exposure represent an important area of research and include both the biological effects and safety levels of these types of radiation. Several studies from in vitro and in vivo research have indicated that these radiations directly affect (positive or negative) biological systems. Figure 1 shows the various sources of EMR with their corresponding frequency ranges, whereas Figure 2 illustrates the various alterations caused by EMR being transmitted into the human body, where these effects can be detrimental, useful, or neutral. This research topic has received significant interest in the last few decades. Specifically, EMR energy absorption by the human body, specifically in the head and neck, has received increased attention recently [28]. Indeed, numerous studies have been conducted on the subject of defining the EMR interactions in biological systems [29,30,31]. Notably, all biological levels are affected by EMR, including microbes, animals, and humans [32]. The physiological alterations to EMR are dependent upon the operating frequencies and peak power. The in-depth mechanisms of microwave bio-interactions have shown that EMR acts as a helping agent to induce genetic changes in biosystems [32]. When studying microwave absorption in animals and humans, various uncertainties remain regarding the relative contributions of indirect heat effects, as well as direct nonthermal interactions to physiological alterations. The biological interactions that occur at the microscopic level are correlated with the dielectric properties of biomacromolecules and bulky molecular units (i.e., cell-membrane receptors and enzyme complexes) [30]. Most cell types contain an electrical gradient (membrane potential) of ~0.1 V across the 40 A° width of the double layer of fat molecules, typically useful for providing an essential structure of a cell membrane [33]. Further, this electrical gradient (105 V·cm^−1^) is believed to be an effective wall against cell stimulation caused by weaker EMRs [33].

### 1.2. Possible Biological Effects and Mechanisms of EMR

**Figure 3:**
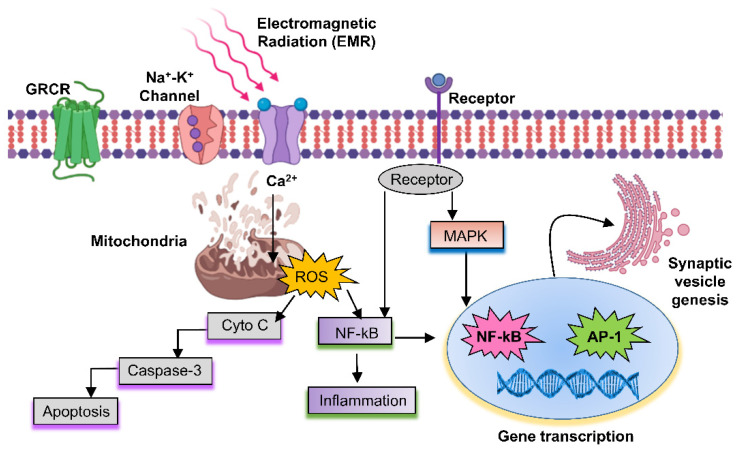
Interactions of RF-EMR with biological systems, and possible mechanisms for inducing various effects. EMR can pass through the membrane using a channel of the cell membrane, and signals that penetrate into the cell produce ROS. Endogenous ROS can activate mitochondrial pathways in apoptosis through caspase-3. In addition, endogenous ROS caused by mitochondrial depolarization activates NF-kB, which causes inflammation. The receptor is one of the EMR pathways that activates the MAPK pathway and then activates target genes such as NF-kB and AP-1 to activate cell death or inflammation.

The precise mechanisms by which microwaves affect the biological system remain largely unknown due to differing equipment and inadequate techniques, bringing uncertainty into the available data. Despite this, many hypotheses have been generated to describe the possible mechanisms of the biological effects of microwaves [34]. An overview of these mechanisms is shown in Figure 2, whereas the most common mechanism is shown in Figure 3. Specifically, microwaves cause electrons or ions to vibrate owing to resonance, which then collide with other molecules inside biological tissues. Opposite charges swing to opposite sides, and charge polarization occurs in the presence of the electric field (EF) provided by the microwave. This polarization is applicable not only for free charges that are irregularly present in the biological tissue, but within individual particles for which the net electric charge is zero as well. Accordingly, this polarization induces the formation of electric dipoles [33]. Continuous repolarization of the induced dipoles and an energy-consuming phenomenon which absorbs the EF energy occurs with the alternating EF. Biological tissues contain large amounts of water [33]. In an alternating EF, the dipoles continually oscillate around their axes, helping to absorb electric energy [35]. Several theories describe the effects of EMR in biological systems [35,36], and an overview is provided in Figure 2. The emission of continuous radio waves (e.g., microwaves) increases the temperatures of the living tissues, whereas nonionizing EMR may also cause biochemical changes that lead to various effects (good, bad, and neutral). Indeed, it is possible that all possible mechanisms depend on the resonance, coherence, and CNS functioning, as well as the stimulation of muscles, reactive oxygen species (ROS), proteins, DNA, and RNA (Figure 2). The input of energy through an external signal may be concentrated when the EMR wavelengths are equal to the molecular energy level differences, resulting in an upsurge in signal strength. Most ions are attached to water, and the dispersion of energy increases system loss when acting on water particles with radio frequencies obtained in resonance. A common concept used to understand the effects of EMR on cells is the induction of supplementary potentials on cellular membranes to interfere with ionic transport [35]. Such changes are only possible when external fields are sufficiently strong, significantly higher than the voltages generated by mitochondrial membranes. Exposure to non-physiological voltage in cell organelles has shown that when the membranes are wider than the cellular membrane, and organelles are comprised of large ionic concentrations, more EMR energy is transmitted through the organelle membrane [35]. This mechanism is used to understand the effects of EMR on cells by inducing variations in molecular bonds that can impact protein enzyme activity [37]. Notably, cellular proteins have diverse structures, and the effects of EMR exposure can vary accordingly [37]. Earlier studies found that protein denaturation, aggregation, and stability can be affected by EMR [38,39], which is why the enzymatic efficiency of a protein is also structure-dependent. Few amino acid side chains in proteins are known to be polar, and respond differently when exposed to different EMR. Microwaves sensitively affect some biological organs in addition to the CNS, reproductive, cardiovascular, and hematopoietic systems. Furthermore, EMR exposure increases reactive oxygen species (ROS) levels inside of tissues [40], leading to macromolecular changes, including DNA/RNA and proteins. This induced oxidative stress increases malondialdehyde, leading to membrane lipid injury, and a reduced glutathione concentration, which plays a key defensive role against various diseases [41].

## 2. Biological Effects of Microwaves

### 2.1. Effect of Microwave on Skin

**Figure 4:**
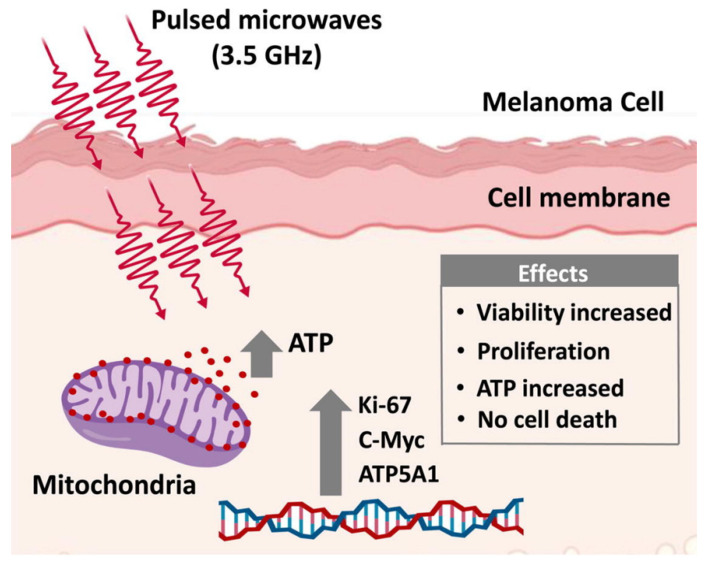
Effects of high-power microwaves (HPM) on skin cancer cells. After exposure of 3.5 GHz HPM, cell death was not induced in skin fibroblasts or melanoma cells. HPM stimulates cell viability and proliferation only in melanoma cells via the expression of genes related to ATP synthesis and proliferation. Reprinted with permission from [45]. Copyright 2020, Elsevier.

Specifically, the effects of microwave radiation on skin are of exceptional importance as it represents the outer layer of exposure to daily radiation. It has been shown that microwaves play a role in inducing cancers on the skin and in the brain tissues [42]. One in vivo study revealed that continuous microwave exposure at a frequency of 10 GHz and a power density of 10 mW·cm^−2^ led to significant changes in molecular markers related to the adaptive stress response in mice skin [43]. Elsewhere, it was shown that microwave exposure at 25 GHz did not induce apoptosis or alterations in pro-survival signaling proteins [44]; however, it did induce an increased number of micronuclei and centromere-positive micronuclei owing to chromosome loss [44]. The effects of pulsed HPM at a frequency of 3.5 GHz on normal skin fibroblast (NHDF) and melanoma (G361 and SK-Mel-31) cells was also studied [45], revealing that HPM did not affect NHDF cells, whereas cell proliferation and increased ATP levels were observed in melanoma (G361) 24 h after exposure (a summary of the results can be seen in Figure 4). Furthermore, 48 h after exposure, the exposed and control groups did not differ significantly, suggesting that HPM exposure functions as a stimulus for skin cancers for up to 24 h [45]. Therefore, it remains crucial to investigate the mechanisms underlying the acceleration of spontaneous and chemically induced cancers with microwave radiation; however, the mechanisms by which micro- and millimeter waves affect the skin remain poorly characterized, and should be further explored [46].

### 2.2. Effect of Microwave on the Reproductive System

**Figure 5:**
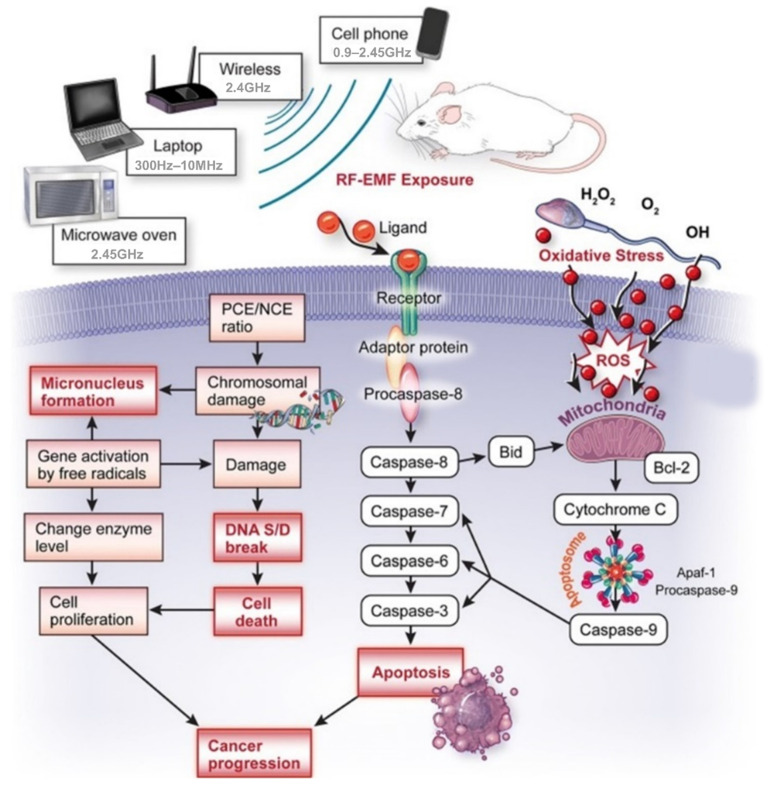
Overview of the available mechanisms displaying the effects of EMR exposure on genotoxic parameters. The mechanisms indicate that EMR-induced oxidative injury/mutation increases the possibilities of DNA damage and micronuclei development while bolstering cancer progression [48].

The use of devices based on EMR, such as phones, Wi-Fi, ovens, radars, and laptops, has increased dramatically. Various studies have reported an association between microwaves and male fertility [47,48,49,50,51,52] (Figure 5 [48]). The human reproductive system has shown adverse effects, and in some cases neutral effects, against various microwaves [48,50]. Specifically, cell phone radiation has also been found to have harmful effects on the male reproductive system [51].

One in vivo study has suggested that microwaves with a frequency of 10 GHz have a deleterious effect on the fertility potential of male rats [52], whereas 2.45 GHz microwave increased inflammation and testicular impairment in the male reproductive system [53,54]. Further, DNA fragmentation has been found to increase following exposure to 850 MHz microwave [55], and was supported by an additional study where DNA fragmentation increased after exposure to 900–1800 MHz microwave radiation [56]. An in vivo study reported that 900 MHz radio frequency radiation activated the p38/JNK-mediated mitogen-activated protein kinase (MAPK) pathway in rat testes [57]. Further, there is strong evidence that the negativity or neutrality of microwave radiation effects on reproductive systems are dependent upon frequency and power. One recent in vivo study investigated the biological effects of 1.5 GHz HPMs on the reproductive system of mice [50], and showed no considerable pathological or ultrastructural changes in testicles, spermatozoa, and serum testosterone levels following 15 min of exposure [50]. Furthermore, no obvious signs of injury or impairment of the reproductive system were found across the bodies of the exposed mice. Alternatively, an increase in the resistance of sperm membranes, as well as a decrease in acrosin activity, number of apoptotic gametes, and seminal plasma PA concentrations, were found following exposure of native human sperm to low-intensity microwave irradiation. Two types of reactions were observed in the sub-fertile samples, and the results revealed the positive bio-effects of specific microwaves on human semen, in addition to the participation of PA in the realization of these effects [58].

Presently, the harmful, neutral, or beneficial effects of microwave radiation on human reproductive abilities cannot be generalized, as it remains possible that certain microwave radiation energy doses are responsible for any of these outcomes. Accordingly, the available evidence and the literature are insufficient for drawing overall conclusions concerning the quantity and forms of microwave energies that produce risk for humans. Further, the current body of evidence provided by animal studies cannot be adequately applied to the human reproductive system, since the cellular membranes of reproductive tissues differ between species. Therefore, it is essential to conduct further studies observing the values of microwave intensity, while obtaining numerical analyses of the energy absorption rate, as microwave radiation poses higher possibilities of different health risks with greater exposure frequencies in both humans and animals.

### 2.3. Effect of Millimeter Range Radiations

Microwaves with millimeter-range wavelengths, known as millimeter-range radiation (MMR), have been investigated as a potential candidate for cancer therapy over the past few decades. MMR has numerous clinical applications [59,60], typically in Eastern Europe, where it can be used to treat >50 diseases, including various forms of cancer. Ultimately, it was claimed that MMR treatment was successful in >3,000,000 patients [61]. One recent in vivo study investigated whether 101 GHz MMR produced by a free-electron laser (FEL) device had toxic effects on healthy mice [61], with the results indicating that all biological parameters were within the normal ranges [61], and no noticeable changes were observed in the physiological, physical, or behavioral statuses of the exposed mice. Furthermore, following exposure, no significant variations were observed in locomotor, exploratory behavior, or anxiety, nor were pathological alterations detected following hematological and biochemical blood analyses [61]; thus, it is concluded that 101 GHz MMRs have no significant toxic biological effects [61]. Alternatively, the primary targets of 60 GHz MMR are typically the eyes and skin [62], where short MMR energy can be absorbed by the cornea, which has a free water content of 75% and thickness of 0.5 mm. Recently, one study assessed the ocular effects caused by 60 GHz MMR [63], with the results showing that MMR did not cause any noticeable physiological alterations [63]. Terahertz (THz) EMR is typically used in security screening, astronomy, tumor imaging, and biomedicine. Further, a recent in vivo study suggested that THz radiation increased antidepression, anti-anxiety, and social interactions in exposed mice [64]. Moreover, THz EMR has been reported to cause death of human primary and malignant cells [65]. At 3.1 THz, exposure can alter the endocytic process of neuronal cells [66]. Although THz EMR has a variety of useful applications for humankind, it remains important to understand its positive, negative, and neutral biological effects, as this field of research should continue to expand [67].

### 2.4. Effect of Microwave Radiation on the Brain

The CNS was determined to be the most vulnerable to microwave radiations [68], with the hippocampus being particularly sensitive [69,70,71,72]. Specifically, microwaves can damage the brain (one of the two key components of the human CNS), particularly affecting the neurotransmitters which play an important role in passing signals inside the body [73]. Accordingly, microwave radiations can cause a delay in the signaling process, resulting in further harmful damage to the body. Conversely, microwaves are extremely useful in the medical field for purposes such as the recognition and diagnosis of tumors at early stages. Overall, microwaves are shown to have positive, neutral, and negative effects on exposed biological systems.

#### 2.4.1. Positive Effects

It is a fact that the advantage of the impact of microwaves on modern life cannot be overlooked [74,75,76,77,78]. Cerebrovascular injuries are a primary cause of physical abnormalities and mortality in many countries. For example, the number of brain strokes can be restricted by minimizing chance, and the identification of appropriate solutions should be prioritized. Stroke causes dynamic electric permittivity of tissues in the brain, which can be detected using microwave tomography [79]. A cold injury to the patient’s hands and feet was successfully treated using microwaves, avoiding amputation [80]. In one study, microwaves were found to have positive effects [81]. Indeed, microwave imaging represents a novel and growing technology for the early diagnosis of various diseases in addition to other positive effects [22,24,82,83,84,85]. For example, recent technologies have been developed for monitoring stroke [86], the diagnoses of lung damage via near-field microwave imaging systems [87], imaging breast cancers [88], and locating tumors and estimating their size [89]. Its use as an alternative, safer imaging technology compared to the present imaging systems (CT and MRI) has also been reported [90]. Moreover, microwave imaging is considered a promising technique for detecting stroke due to its low costs, intrinsic contrast mechanism, and short relatively acquisition time [91]. Indeed, a compact microwave-based scanner for stroke detection and management was designed [92] for use in an ambulatory mode. One in vivo study indicated that phospholipid and triglyceride metabolism was significantly modified by 2.856 GHz microwave exposure in rats [93], whereas microwave exposure at a frequency of 1.8 GHz significantly increased the permeability of ^14^C-sucrose [94]. Elsewhere, microwaves at 800–1000 MHz have been shown to induce a significant adaptive survival response [95]. The detection of tumors and early-stage cancers using microwaves is one of the most promising methods for diagnoses of serious tumors at early stages. Accordingly, microwave imaging is an attractive screening technique that is rapidly increasing in the field of research, as this technique offers some encouraging advantages, such as patient comfort, affordability, nonionizing nature, and noninvasiveness, compared to X-ray mammography, ultrasound, or MRI. However, the safety of microwave imaging remains a critical unknown, requiring further investigation.

#### 2.4.2. Neutral Effects

As there is a wide range of microwave-based applications in daily life, its neutral effects on humans would be considered a positive sign. Indeed, numerous studies have shown evidence of the neutral effects of microwave radiations with different frequencies, energy doses, and exposure times. Specifically, high-frequency microwave (5.8 GHz) has attracted interest for applications in wireless technology. For example, one study observing its biological effects found that microwave exposure at a frequency of 5.8 GHz had no obvious effects on hippocampal synaptic plasticity, learning, and memory ability in rats [96]. Another in vitro study reported that exposure to 5.8 GHz microwave radiations had little to no effect on genotoxicity [97]. Elsewhere, it was found that in vitro exposure to 935 MHz microwave radiations did not cause apoptosis of microglial and SH-SY5Y cells [98]. Overall, a large body of evidence exists in which it has been observed that microwave radiations have no obvious effects on different organs; thus, it remains necessary to standardize the parameters in which microwave neutrality on humans can be defined, and the development of microwave-based technologies should consider the frequencies that have neutral effects on biological systems.

#### 2.4.3. Negative Effects

**Figure 6:**
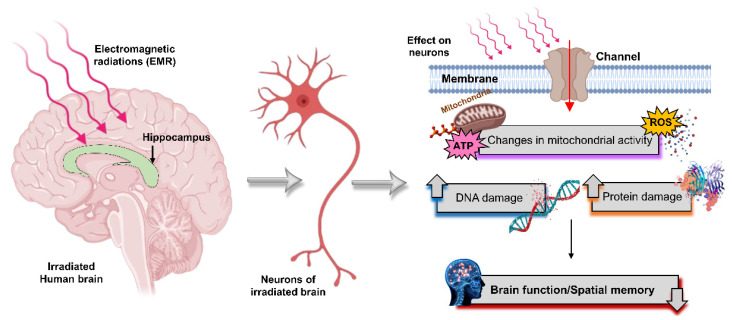
Representation of microwave radiation effects on the brain. Microwave radiation induces protein damage in neurons, changes mitochondrial activity by influencing the formation of ROS and ATP levels, and causes breaks in single- and double-strained DNA, which leads to brain dysfunction and declines in spatial memory.

Numerous further studies have revealed the harmful effects of microwaves on the human brain, and a diagram of the most common deleterious effects of microwave radiation neurons in the brain is shown in Figure 6. As stated, effects vary with the operational frequency, microwave intensity, and exposure time. For example, there are reports showing that microwave exposure causes DNA damage [99,100,101], which is directly associated with health hazards, as DNA damage inside neurons can lead to neurodegenerative diseases. With the increasing number of microwave-based applications across various operational frequencies and powers, biological studies are essential to determining the optimal parameters. Notably, cell phone radiation penetrates the body at frequencies of 1800 and 2100 MHz, and can induce oxidative stress, leading to DNA strand breaks and liver damage in exposed rats [100]. Elsewhere, cell phone radiation at 900, 1800, and 2100 MHz has been correlated with increased DNA damage and lipid peroxidation in rats [102], whereas cell phone radiation at 2400 MHz can affect the hippocampal structural integrity, leading to behavioral changes (e.g., anxiety) [103,104]. Further, it has been shown that 2.45 and 16.5 GHz exposure can cause considerable DNA single-strand breaks in vivo [105], while DNA viscosity was reduced when exposed to X-rays owing to their molecular weight after DNA breaks [106,107]. Moreover, some studies found that microwaves can produce different biological effects on the CNS, and may be involved in the occurrence of CNS diseases [108], including Alzheimer’s disease [109]. Accordingly, a study was conducted confirming that exposure to 2.45 GHz microwaves caused damage to an exposed rat brain, leading to the loss of memory and decline in learning abilities [110].

In another study, it was revealed that caspase-3 was triggered by 2.45 GHz exposure [110]. Elsewhere, it was shown that as a result of oxidative and nitrosative stress induced by microwave radiation, hippocampal neuronal and non-neuronal apoptosis occurred, while p53 was overexpressed as a result of upregulation of Bax and downregulation of pro-caspase-3 along with poly ADP-ribose polymerase (PARP) 1, resulting in neuronal degeneration through apoptosis [111]. Furthermore, microwave radiation induced autophagy in rat hippocampal neurons at specific levels, while excessive autophagy may be detrimental by facilitating synaptic vesicle destruction and impairing synaptic plasticity [112]. A distinct study found that exposure to 1.5 and 4.3 GHz microwaves induced cognitive impairment and damage to hippocampal tissues in vivo, where combined frequency exposure was found to have more damaging effects than a single exposure [113]. Notably, these findings offer insight into observing the combined effects of different frequencies that are commonly present in the atmosphere. Elsewhere, recent in vivo and in vitro studies have reported that microwave radiation causes autophagy in neuronal cells by activating the miR-30a/AMPKα2 pathway [71]. Furthermore, evidence supports the presence of nonthermal effects and exposure difficulties for the blood barrier of the brain [114], with 1.5 and 2.856 GHz microwave radiation exposure inducing a decline in spatial memory [115]. Similarly, a 10 GHz microwave exposure was shown to impair spatial memory, enzyme activity, and histopathology of mouse brains [116], while it has also been reported that neural cells are damaged (apoptosis) via the mitochondria-dependent caspase-3 pathway [117]. Several studies assessing the positive, negative, or neutral biological effects of microwaves based on different frequencies are summarized in Table 1.

Free radicals are reactive molecules generated during the conversion of foodstuff into energy by oxygen, the production of which is oxygen-dependent [118]. As oxygen is essential for life, the generation of free radicals inside the body is unavoidable. External factors, such as microwave radiation, can alter the translation and transcription of genes via the epidermal growth factor receptor, leading to the excessive or overproduction of ROS [119,120,121]. For example, the Fenton reaction is a well-known catalytic process converting hydrogen peroxide (mitochondrial oxidative respiration) into toxic hydroxyl free radicals, and numerous studies have reported that microwave radiation represents another important mechanism of this reaction, indicating that it can promote free radical activity in cells [120,122]. Further evidence provided by scientists shows that ROS play a beneficial role against cancers, although high ROS production by microwave radiations may damage brain cells when microwave-radiation-produced radicals react with biomolecules in the brain to change their activity or DNA (Figure 6).

**Table 1:** Effects of different microwave frequencies on brain.

Ref. No.	Frequency	Study Type	Main Findings	Effects
[123]	2.45 GHz	in vivo	Irradiated rats showed a significant decrease in spatial learning and memory performance.	Negative
[111]	2.45 GHz	in vivo	Microwave exposure led to oxidative/nitrosative stress that induced p53 activation of hippocampal neuronal and nonneuronal apoptosis related to memory loss.	Negative
[124]	0.9, 1.8, and 2.45 GHz	in vivo	Microwaves decreased cognitive functions while increasing HSP70 levels and DNA damage in the brain.	Negative
[105]	2.45 and 16.5 GHz	in vivo	Microwave exposure caused DNA single-strand breaks.	Negative
[125]	0.9 GHz	in vitro	No obvious changes were observed in promyelocytic leukemia (HL-60) and neuroblastoma (SK-N-SH) cell lines following microwave exposure.	Neutral
[98]	0.935 GHz	in vitro	No effects in murine microglial (N9) and human neuroblastoma (SH-SY5Y) cells following microwave exposure.	Neutral
[126]	0.9 GHz	in vitro	Increased apoptotic sub-G1 DNA content in human neuroblastoma (SH-SY5Y) cells. Short-term exposures induced a transient rise in Egr-1 mRNA levels, along with activating MAPK subtypes ERK1/2 and SAPK/JNK.	Negative
[127]	0.8–0.9 GHz	in vivo	Microwave exposure led to significant epigenetic modulations in the hippocampus.	Negative
[128]	2.856 GHz	in vivo	Rats exposed to 10 and 50 mW·cm-2 microwaves showed a significant decrease in spatial learning and memory, whereas 5 mW·cm-2 showed no change.	Negative
[93]	2.856 GHz	in vivo	Phospholipid and triglyceride (TG) metabolisms were significantly modified in exposed rats.	Positive
[129]	2.856 GHz	in vivo, in vitro	Microwave exposure at 30 mW·cm-2 altered synaptic structure, amino acid release, and calcium influx.	Negative
[130]	1.7 GHz	in vitro	No effects on human-adipose-tissue-derived stem cells (ASCs) or liver cancer stem cells (Huh7) following microwave exposure.	Neutral
[131]	1.8 GHz	in vitro	Microwave exposure may have decreased the excitatory synaptic activity and the number of excitatory synapses in rat hippocampal neurons.	Negative
[132]	1.8 GHz	in vivo	Hippocampi were injured by long-term microwave exposure, leading to the impairment of cognitive function owing to neurotransmitter disruption.	Negative
[133]	1.8 GHz	in vitro	Microwave exposure at indicated frequencies during the early developmental stage may have influenced dendritic development and excitatory synapse formation in hippocampal neurons.	Negative
[94]	1.8 GHz	in vitro	Microwave exposure significantly increased permeability for ^14^C-sucrose.	Positive
[134]	1.9 GHz	in vitro	No significant changes were observed across three human-derived immune cell lines (HL-60, Mono-Mac-6, TK6) following microwave exposure.	Neutral
[95]	0.8–1 GHz	in vitro	Microwave radiation exposure across a given frequency range may have induced a considerable survival adaptive response.	Positive
[135]	1 GHz	in vitro	Microwave radiation did not influence efflux in rat brain tissue.	Neutral
[136]	9.3 GHz	in vivo	Irradiation did not affect neuron ability, as no lasting or delayed effects were observed at the analyzed frequency.	Neutral
[69]	50 GHz	in vivo	Microwave exposure caused DNA double-stranded breaks, and changed antioxidant enzymes in the neurological system due to free radical formation.	Negative
[96]	5.8 GHz	in vivo	Microwave exposure did not show any obvious effects on the hippocampal synaptic plasticity of the selected rats at the indicated frequencies.	Neutral
[97]	5.8 GHz	in vitro	Microwave exposure had little to no effect on DNA strand breaks, micronucleus formation, and Hsp expression in eye cells at the assessed frequencies.	Neutral

### 2.5. Protective Techniques to Treat Cancers

#### 2.5.1. Nonthermal Atmospheric Pressure Plasma

**Figure 7:**
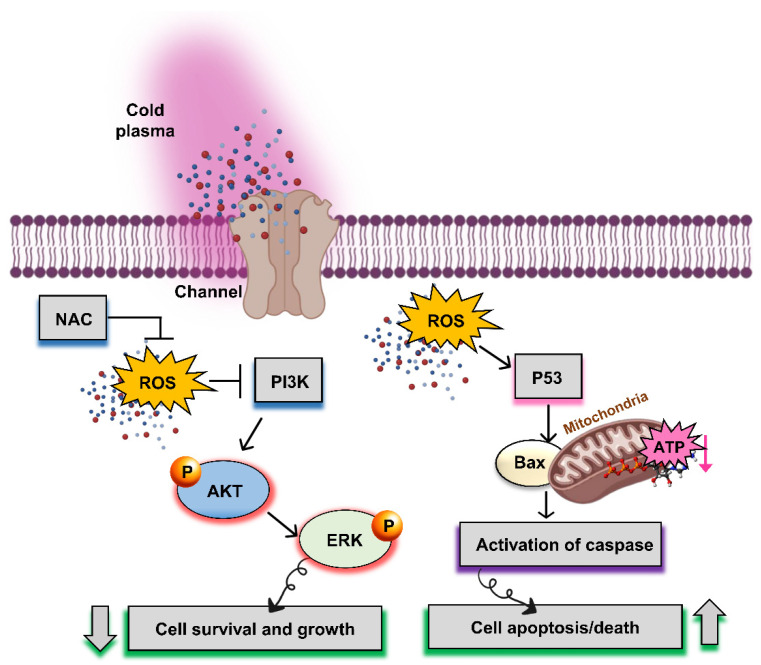
Graphical representation of cellular mechanisms underlying nonthermal plasma in a brain tumor to inhibit its growth and increase cell apoptosis. The PI3K signaling pathway promoting cell survival and growth is involved in the apoptosis of the central nervous system. AKT or PKB is a primary protein effector downstream of the PI3K signaling pathway. ERK plays an important role in the growth mechanism by regulating the PI3K/AKT pathway. ROS produced by nonthermal plasma are also involved in the pathophysiological process of apoptosis in brain tumors. The effect of p53 on caspase regulation depends on the mitochondrial mechanism. Thus, Bax stimulates the release of cytochrome c and activation of caspase signaling, thereby increasing cell apoptosis and cell death.

Recently, nonthermal biocompatible atmospheric-pressure plasma (i.e., cold plasma) has provided a new horizon for various prospective biomedical applications [137,138,139,140,141,142], and represents a promising technique for the treatment of cancers and tumors without harming normal cells or tissues. Over the past few decades, “plasma medicine” has been used for its anticancer properties [143,144,145,146,147,148], including one clinical study [149]. Plasma is such a powerful tool because of its advantageous properties in the field of medicine, such as inactivation of microorganisms, dental uses, skin rejuvenation, and cancer therapy [143,150]. Nonthermal plasma has a wide range of applications in modern age in several areas [11,151,152,153]. It comprises multiple energies in the form of electrons, ions, and reactive oxygen and nitrogen species (RONS) [154,155,156,157], for which cocktails of the latter have been shown to have apoptotic properties in cancer cells [140,158]. As cancer cells maintain different mechanisms to normal cells, it has been reported that ROS uptake by cancer cells induces apoptosis via intrinsic or extrinsic pathways in brain tumors. The cellular mechanisms underlying nonthermal plasma in brain tumors are shown in Figure 7.

**Figure 8:**
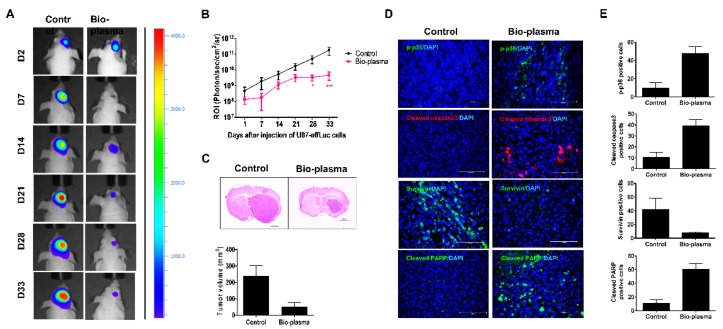
Targeting a brain tumor via nonthermal plasma in vivo, showing (**A**) bioluminescence imaging; (**B**) the region of interest levels for tumor changes following plasma treatment; (**C**) sectioned mouse brain tumor volume and size determined by bioluminescence imaging; and (**D**,**E**) expressions of cleaved caspase-3, p-p38, cleaved poly (ADP-ribose) polymerase (PARP), and survival, as determined by immunofluorescence [158].

It is well established that U87 MG is a highly sensitive brain cancer cell line [159]. Recent studies have suggested that cold plasma plays a significant role in U87 MG [160], specifically via the total mitogen-activated protein kinase (MAPK) signaling pathway [140,158] without affecting normal cells. Indeed, it was observed that the survival rates in plasma-treated groups were much higher than those in nontreated groups, in addition to a notable reduction in tumor size in mice (Figure 8) [158]. Therefore, this study demonstrated the existence of nonthermal effects, as well as exposure complexities, that must be taken into account for a thorough evaluation and assessment of potential health effects necessary for future studies. Following treatment with nonthermal plasma, higher concentrations of RONS were present inside cancer cells compared to normal cells. Accordingly, further challenges related to dealing with additional oxidative damage from RONS present in the plasma were created, while healthy cells were better able to protect themselves. Elsewhere, it has been confirmed that nonthermal atmospheric pressure plasma is a more powerful tool for inducing apoptosis in brain cancer cells through the generation of RONS. In both in vivo and in vitro studies, treatment time was a central factor in the inhibition of cancers. As tumor inhibition is facilitated by cell cycle arrest, the treatment period is important for defining cytotoxicity and apoptosis levels. To this end, a growing body of evidence confirms that plasma treatment induced cell death, while morphological changes, cell cycle arrest, and apoptosis genes were all increased in cancer cell lines [139,143,158,161,162,163,164]. These outcomes directed the anticancer activity of nonthermal plasma, offering potential for curing future cancers without affecting normal and healthy tissues. Furthermore, it may also be effective for healing wounds. Accordingly, it was concluded that plasma serves an array of important roles, and will likely provide many significant benefits for humans in the future.

#### 2.5.2. Flavonoids

Both natural and artificial EMRs are widespread in the environment. To this end, protective techniques should be developed with an increasing number of EMR-based applications and devices in the near future, as currently available techniques are insufficient to overcome the potentially harmful effects of ever-increasing doses of radiation [165,166,167]. As covered in Section 2.4, microwave radiation can modulate responses in the CNS, where higher doses can produce ROS, oxidative stress, and neuroinflammation [120,167,168]. Treatment with natural products is, thus, necessary to reduce these harmful effects [169].

**Figure 9:**
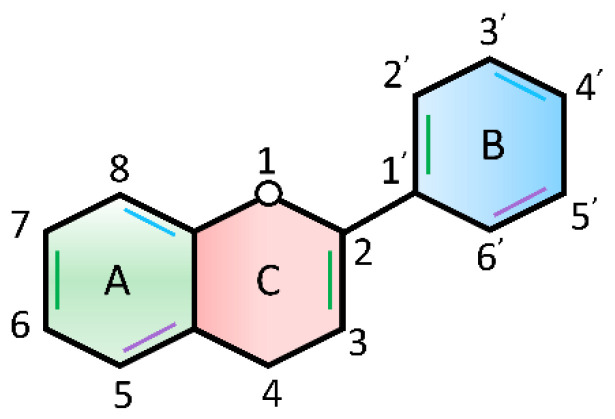
Basic flavonoid structure.

**Figure 10:**
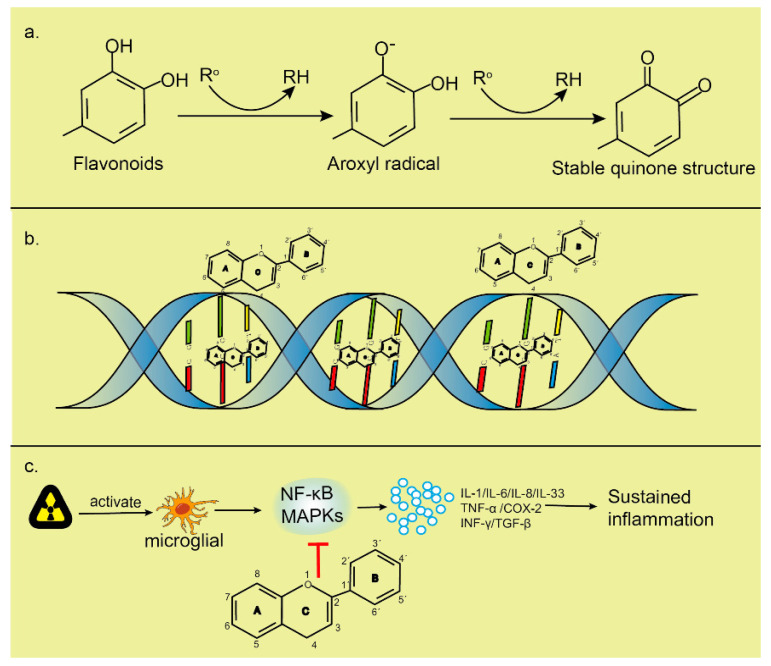
Possible mechanisms of flavonoids within the biological system: (**a**) decreasing highly oxidized free radicals with redox potentials via the donation of a hydrogen atom. Ro indicates the peroxyl, superoxide anion, hydroxyl, and aroxyl radicals, where the latter may react with the second radical to produce a stable quinone structure; (**b**) intercalation of flavonoids with DNA double helices maintains the helical structures and condensation of DNA in an extremely compact form that is less vulnerable to attack by existing free radicals. Flavonoids can interact with the phosphate moiety of the DNA backbone through hydrogen bonding, thereby repairing sugar radicals; and (**c**) preventing the activation of NF–κB and MAPK apoptotic pathways, playing an anti-inflammatory role by reducing the release of inflammatory factors [170].

One possible treatment lies in flavonoids, which are chemically based on a fifteen-carbon skeleton containing two benzene rings (Figure 9A,B), linked via a heterocyclic pyrene ring (Figure 9C) [170,171,172]. Flavonoids are believed to be effective antioxidants capable of inhibiting transcription factors or the regulatory enzymes necessary for controlling inflammatory mediators, affecting oxidative stress through DNA interactions, and increasing genomic stability. Several studies have shown that flavonoids not only enhance the radiosensitivity of cancer cells, but also protect normal tissues from EMR-induced damage [169,173,174]. Flavonoids are also anti-inflammatory scavengers of free radicals. Flavonoids are plant-derived compounds that exist naturally in various Chinese medicines, and exhibit radioprotective and neuroprotective properties [175,176,177,178]. Both flavonoids and their metabolites can cross the blood–brain barrier [179], which is composed of capillary endothelial cells, basement and neuroglial membranes, and glial podocytes [180], and approach brain cells to decrease brain injury, while improving neurodegenerative diseases and cognitive impairment [181,182,183]. The neuroprotective mechanisms of flavonoids may include antioxidation, anti-apoptosis, reduction of inflammation in the CNS [184], and the regulation of various intracellular and extracellular targets [185] (Figure 10).

## 3. Discussion

Modern progress in innovative technologies has led to EMRs covering nearly every aspect of human life. Currently, microwaves are being studied for their potential in therapeutic imaging and detection of early-stage tumors in the medical field [16,17,18]. With advances in electronics and novel microwave-based systems, microwave radiation has become an indispensable part of modern life, while avoiding its exposure is near impossible. The ever-present exposure raises significant concerns surrounding the biological effects and safety levels of microwave radiation exposure. To this end, a growing body of evidence from in vitro and in vivo studies has found that microwave radiation can negatively, positively, or neutrally affect biological systems based on their physical parameters. When microwave radiation interacts with a biological system, it causes various changes (Figure 2). These changes may be harmful, useful, or neutral in the living body [28,186]. To date, the effects of microwave radiation have been observed at the microbial cell level in both animals and humans [32]. Among them, HPM-based technologies have been increasing rapidly, particularly to expand the detection range of radars. Notably, HPMs showed no deleterious effects on normal skin fibroblast cells, but increased viability and ATP levels were observed in melanoma immediately following exposure [45], whereas all changes returned to nonsignificant levels by 48 h after exposure. These findings suggest that 3.5 GHz HPM exposure can function as a stimulus for skin cancers up to 24 h only at higher doses, while the exposure of skin cancer patients to HPM (3.5 GHz) should be limited [45]. Although it is presently not possible for humans to avoid all microwave exposure, they should be aware of the possible biological threat. Additionally, microwave exposure changed reproductive endocrine hormones, embryonic development, gonadal function, pregnancy, and fetal progression [187]. Moreover, the CNS of the biological system is thought to be the most vulnerable to microwaves, especially the hippocampus [69].

Tumor-treating fields (TTF) therapy, which uses low-intensity (1–3 V/cm), intermediate-frequency (100–300 kHz) alternating electric fields to target tumors, was first proposed as a cutting-edge cancer treatment option in 2004 [188,189,190]. Preclinical results show that TTF has additive or synergistic action with chemotherapy and an antimitotic impact that is intensity- and frequency-dependent [188]. TTF is a potential new anti-invasion and anti-angiogenesis therapy method that has recently been demonstrated to be effective in treating patients with glioblastoma multiforme [191]. Due to higher glycolysis, ion concentration, and permittivity in malignant compared with nonmalignant tissues, the electric field and the ensuing heat (electrohyperthermia) can together promote cell death in tumor tissue [192]. Cancer treatment with hyperthermia permits tumor masses to reach temperatures between 39 °C and 43 °C. Fourteen of the most pertinent publications demonstrating the advantages of hyperthermia were chosen from 1294 articles that were recently reviewed and evaluated [193]. Additionally favorable benefits are seen when immunotherapy is used in combination with heat therapy [194,195,196]. It is often used as a supplemental therapy, frequently in conjunction with radiation and/or chemotherapy to boost the efficacy and extend their therapeutic advantages [197]. A recent study showed that the modulated electrohyperthermia may improve tumor response and survival of pancreatic cancer patients [198].

Interestingly, an EF of ~11 kV·cm^−1^ [27] was found to be approximately similar to the EFs of a conventional nonthermal atmospheric pressure plasma jet [155,199]. EFs interact with molecular nitrogen and oxygen, converting them into atomic species which are further combined to form NOx that is incorporated into the liquid [27]. Increased intracellular ROS levels were also observed in response to HPM exposure, which led to lethal bacterial damage [26]. These findings clarify the mechanisms of HPM-specific effects on bacterial cells and their biomolecules, which can help establish safety standards for HPM exposure without any heating effects on different organisms. Sterilization via HPM is another effective method, as all kinds of bacteria can be killed using HPM energy under lower temperatures and shorter times compared with traditional sterilization techniques [200]. Moreover, HPM sterilization offers several benefits and potential applications across various fields.

**Figure 11:**
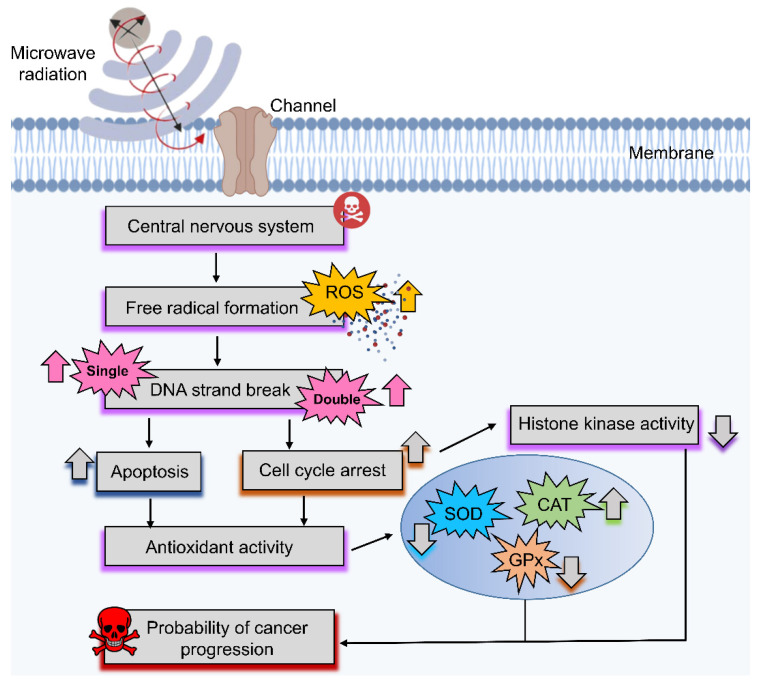
Overview of the most common effects of microwave on the central nervous system (CNS), notably increasing free radical formation and DNA single- and double-strand breaks, as well as cell cycle arrest and apoptosis, leading to elevated probability of cancer progression in the brain.

Microwaves have several useful applications in the medical field [201]. For example, microwave imaging is the best method for diagnosing serious tumors at an early stage; however, certain frequencies and exposure times can be harmful to biological systems. Accordingly, establishing safety levels by standardizing the frequency, power, and exposure times of microwaves, along with their applications, is of the upmost importance. Some studies have reported that microwaves may affect our CNSs differently, with some indicating that microwaves are involved in the manifestation of CNS diseases [108], including Alzheimer’s disease [109,202,203,204,205,206]. (Figure 6). Microwaves have been shown to cause damaging alterations in rat brains at frequencies of 2.45 GHz (a typical frequency of microwave ovens), leading to the loss of memory and learning abilities [110]. Furthermore, caspase-3 was triggered by 2.45 GHz microwave radiation, inducing oxidative and nitrosative stress, and leading to hippocampal neuronal and non-neuronal apoptosis [111]. Microwave radiation effect on biological systems [105], especially the CNS via the formation of ROS, in addition to DNA single-and double-strand breaks, can enhance the probability of cancer progression (Figure 11). The operational frequency of EMR, including intensity, and exposure time are key determining factors of different biological effects (positive, negative, or neutral). Microwave radiation has been shown to activate autophagy in rat hippocampal neurons at certain EMR energy doses, although excessive autophagy may be harmful by damaging synaptic plasticity via mediating synaptic vesicle degradation [112]. Notably, mitochondrial injury occurs earlier and more severely in the brain than other organs with radiation exposure [207]. In biology, mitochondria play a significant role by providing energy in the form of ATP, yet microwave radiation can cause several metabolic disorders within these organelles [208]. Accordingly, several key factors should be evaluated in future prospective microwave studies. Mitochondrial damage plays a significant role in inducing several apoptotic markers inside cells, which provide signals for apoptosis. In cancer cells, apoptosis is considered a positive outcome, but when induced in normal healthy cells, it can cause remarkable future damage. Further, microwave damage is sometimes associated with neurodegenerative disorders; for example, Alzheimer’s disease requires further analysis [109]. As a key component of the CNS in the human body, microwaves can have adverse effects on the CNS deleteriously affecting the brain, including neurotransmitters, which play a key role in passing signals throughout the human body. Accordingly, microwave-induced injury to neurotransmitters delays the signaling process, causing harmful damage to the body. Therefore, it is essential to develop safety techniques or useful methods for reducing the harmful effects, or protecting against these radiations as the number of microwave-based applications continues to increase.

Nonthermal atmospheric pressure plasma or plasma medicine is a relatively modern field of research for application in bioengineering, with cancer therapy representing one of its most promising applications. Multiple studies have found that plasma treatment induces morphological changes, cell cycle arrest, and the induction of apoptotic genes in cancer cells following plasma treatment [139,143,168]. Notably, this field of research is growing rapidly. Over the past few decades, “plasma medicine” has been employed for its anticancer properties [143,144,145,146,147], which have also shown to be successful in clinical studies [149,209,210,211,212]. These outcomes are focused on the anticancer activity of nonthermal plasma, providing new potential methods for curing serious cancers without affecting normal and healthy tissues in the future. Additionally, treatment with natural products extracted from medicinal plants and safe techniques to minimize potential harm to healthy organs are important. Flavonoids represent one such group of plant-derived compounds displaying important radioprotective and neuroprotective properties while reducing DNA damage and inflammation within the CNS [175]; thus, flavonoid treatment may be an important and promising therapeutic alternative for avoiding radiotherapy-induced pathophysiological alterations in the brain, as well as cognitive impairment.

## 4. Conclusions

With advances in electronics and novel microwave-based systems, microwave radiation has become an indispensable part of modern life, while avoiding its exposure is nearly impossible. Humans are swimming, similar to fish, in a vast ocean of different radiations in this environment, resulting in frequent exposure. As a result, studying the biological impacts of these radiations has become an important subject of study. Microwave radiations have positive, negative, and neutral effects, which are highly dependent on EM field strengths, operational frequencies, and exposure times. With advancements in medical technologies, microwaves have played a major role in the treatment and detection of early-stage tumors; however, they can also have adverse effects on the CNS, including neurotransmitters, which play a key role in passing signals inside the human body. Accordingly, microwave-induced injury to neurotransmitters can cause a delay in the signaling process, which has critical implications for body function. Both natural and artificial microwave radiations are widespread in the environment. To this end, protective techniques should be developed equally to serve humanity. Cold plasma is such a powerful tool because of its advantageous properties in the field of medicine, such as inactivation of microorganisms, dental uses, skin rejuvenation, and cancer therapy for treating cancers/tumors without affecting healthy tissues. Flavonoids are plant-derived compounds that exist naturally and exhibit radioprotective and neuroprotective properties. The flavonoids also represent new and emerging technology for treating cancers/tumors without affecting healthy tissues.

Further, it is essential to consider the health effects of specific frequencies when advancing microwave-based applications, while new approaches and several factors require further experimental evaluation to establish correlated safety standards by optimizing positive effects and minimizing harmful effects.

## Data Availability

Not applicable.
